# Cattle co-infection of *Echinococcus granulosus* and *Fasciola hepatica* results in a different systemic cytokine profile than single parasite infection

**DOI:** 10.1371/journal.pone.0238909

**Published:** 2020-09-11

**Authors:** Felipe Corrêa, Christian Hidalgo, Caroll Stoore, Mauricio Jiménez, Marcela Hernández, Rodolfo Paredes

**Affiliations:** 1 Escuela de Medicina Veterinaria, Facultad de Ciencias de la Vida, Universidad Andres Bello, Santiago, Chile; 2 Department of Companion Animal Clinical Studies, Faculty of Veterinary Science, University of Pretoria, Pretoria, South Africa; 3 Instituto de Ciencias Agroalimentarias, Animales y Ambientales (ICA3), Universidad de O’Higgins, San Fernando, Chile; 4 Laboratorio de Biología Periodontal y Departamento de Patología y Medicina Oral, Facultad de Odontología, Universidad de Chile, Santiago, Chile; Centro de Investigacion y de Estudios Avanzados del Instituto Politecnico Nacional, MEXICO

## Abstract

*E*. *granulosus* is a cestode that causes Cystic Echinococcosis (CE), a zoonotic disease with worldwide presence. The immune response generated by the host against the metacestode induces a permissive Th2 response, as opposed to pro-inflammatory Th1 response. In this view, mixed Th2 and regulatory responses allow parasite survival. Overall, larval *Echinococcus* infections induce strong regulatory responses. *Fasciola hepatica*, another common helminth parasite, represents a major infection in cattle. Co-infection with different parasite species in the same host, polyparasitism, is a common occurrence involving *E*. *granulosus* and *F*. *hepatica* in cattle. ‘While it is known that infection with *F*. *hepatica* also triggers a polarized Th2/Treg immune response, little is reported regarding effects on the systemic immune response of this example of polyparasitism. *F*. *hepatica* also triggers immune responses polarized to the Th2/ Treg spectrum. Serum samples from 107 animals were analyzed, and were divided according to their infection status and *Echinococcal* cysts fertility. Cytokines were measured utilizing a Milliplex Magnetic Bead Panel to detect IFN-γ, IL-1, IL-2, IL-4, IL-6, IL-10, IL-12 and IL-18. Cattle infected only with *F*. *hepatica* had the highest concentration of every cytokine analyzed, with both 4.24 and 3.34-fold increases in IL-10 and IL-4, respectively, compared to control animals, followed by *E*. *granulosus* and *F*. *hepatica* co-infected animals with two-fold increase in IL-10 and IL-4, compared to control animals, suggesting that *E*. *granulosus* co-infection dampens the cattle Th2/Treg immune response against *F*. *hepatica*. When considering *Echinococcal* cyst fertility and systemic cytokine concentrations, fertile cysts had higher IFN-γ, IL-6 and IL-18 concentrations, while infertile cysts had higher IL-10 concentrations. These results show that *E*. *granulosus* co-infection lowers Th1 and Th2 cytokine serological concentration when compared to *F*. *hepatica* infection alone. *E*. *granulosus* infections show no difference in IFN-γ, IL-1, IL-2, IL-6 and IL-18 levels compared with control animals, highlighting the immune evasion mechanisms of this cestode.

## Introduction

Cystic Echinococcosis (CE), formerly known as hydatid disease, is a zoonosis with worldwide distribution [1, 2]. It is caused by *Echinococcus granulosus sensu lato* metacestodes. The metacestode, usually called hydatid cyst but currently termed *Echinococcal* cyst, is found in a wide range of mammals such as cattle and sheep [3], while humans act as dead-end hosts [4]. This parasite has an indirect life cycle, with canids such as dogs participating as definitive hosts, with the adult worm living in the small intestine and eliminating gravid proglottids and eggs with the feces, which are later ingested by the aforementioned herbivores, who participate as intermediate hosts [5]. *Echinococcal* cysts develop within the internal organs (frequently liver and lungs) of intermediate hosts as unilocular fluid-filled bladders, where protoscoleces are formed [6]. Finally, when a definitive host ingests viable protoscoleces, they evaginate and attach to the small intestine, developing the adult parasite and completing the cycle [7].

Within the viscera of intermediate hosts, *Echinococcal* cysts are able to survive by evading the host immune response [8]. Research in human CE patients and secondary CE mouse models indicate that during chronic infection, the immune responses has mixed T helper 1 (Th1), T helper 2 (Th2) and T regulatory (Treg) profile, which plays an important role in promoting the progression of the disease by the secretion of both IFN-γ and IL-4 [9–11].

The biological importance of a polarized cytokine response is seen in some infectious diseases where Th1 or Th2 responses are correlated with susceptibility or resistance [12, 13]. Understanding what drives cytokine responses towards different expression patterns is relevant for the rational design of immune intervention, possible vaccination protocols and diagnosis. In fact, parasite immune modulation can interfere with diagnostic tests, such as in *Mycobacterium* and *Fasciola* co infections [14, 15]. Polarization of cytokine response is a complex phenomenon which is affected by the conditions present during T-cell activation, such as cytokines, co-stimulation, strength of TCR signaling, immunomodulatory molecules produced by parasites and the chemical nature of antigens [12, 16].

Cattle immune responses to CE follows the same pattern of mixed immune response with, an early Th1 response with a later Th1, Th2, and Treg mixed response, which is responsible for CE chronic infection [17]. In chronic parasitic infections, parasites express effective immune evasion mechanisms. This is particularly noted as EC’s often live several years and grow to a large size within the host [18].

It was previously understood that *Echinococcus* cyst immune evasion was due to pure Th2 instead of a Th1 response [19, 20]. It is now accepted, through other studies, the evasion is achieved by a combination of Th2 and Treg responses [21, 22]. This type of response, called Th2-like response in some publications, promotes parasite survival in the intermediate host [18]. In human parasitic infections the host utilizes several innate and acquired protective mechanisms but not all of these responses are effective. A particular characteristic of CE is chronic infections persist with detectable humoral and cellular responses. Different antigens are expressed during different stages. The human host responds independently to a myriad of antigenic stimuli; the invading oncosphere, the metacestode in transformation from the oncosphere, and finally the mature metacestode [23].

Two kinds of *Echinococcal* cysts are found In naturally infected intermediate hosts; (1) fertile cysts, in which protoscoleces are attached to the germinal layer or free into the hydatid fluid; and (2) infertile cysts, where protoscoleces are absent and the parasite life cycle ends [24]. The reason why these two type of cysts exist is unclear [25]. The host immune response is likely to participate in generating infertile *Echinococcus* cysts [24]. However, the parasite antigen that drives this immune response remains unknown.

Previous reports indicate that 71.5–91.2% [26–29] of cattle *Echinococcal* cysts are infertile [29], implying that cattle are an important source of infection to the definitive hosts of this parasite, as 8.8–28.5% of cysts are fertile. Regarding the metacestode tropism, the lung is the most common organ for the development of fertile cysts [26, 30]. Fertile and infertile cysts are caused by the same haplotype of *Echinococcus granulosus sensu stricto* [24], suggesting that parasite genetics alone cannot explain the fertility of *Echinococcus* cysts.

Since most data of cyst fertility comes from naturally infected cattle slaughtered at abattoirs, the hosts are usually infected with other parasites besides *Echinococcus granulosus*, a condition known as polyparasitism. This condition may affect the host susceptibility to other infections, some are fatal [31–34]. Although extremely relevant for the comprehension of the host-parasite relationship, there is scarce information in the literature that allows determination of the real effects of polyparasitism in host health [35]. A previous study indicates that polyparasitism between *Echinococcus granulosus* and *Fasciola hepatica* is associated with a decrease in *Echinococcal* cysts found in liver by CE and, an increase in lung *Echinococcal* cysts, particularly small cysts [36]. There are no differences between B and T cell infiltration in the adventitial layer of both fertile and infertile cysts when the host is co-infected with *Fasciola hepatica* [37].

*Fasciola hepatica*, also a platyhelminth, represents a major infection in cattle named fascioliasis. A study of *Echinococcus granulosus* and *Fasciola hepatica* co-infection showed that it can happen in almost 10% of cattle [36]. As with other helminths, *Fasciola hepatica* infection triggers a mixed Th2/Treg immune response [38]. Both *E*. *granulosus* and *F*. *hepatica* induce a chronic infection surviving inside its mammalian host for more than a decade [6, 39].

The purpose of this study was to explore the effect of *Fasciola hepatica* polyparasitism on the systemic cytokine profiles in serum from cattle with both fertile and infertile *Echinococcal* cysts. This co-infection is common but reports related to serum cytokine profile are not found in current literature.

## Materials and methods

### Sampling

The Universidad Andres Bello Bioethics Board approved the study protocol (protocol number 016/2016). One slaughtering-day visit per week was scheduled. Official abattoir veterinarian inspectors examined the animals before the slaughter and were deemed healthy for slaughtering. After slaughter, internal organs (lungs, liver, heart, spleen and kidney) of slaughtered cattle where thoroughly examined for the presence of *Echinococcus granulosus sensu lato* and *Fasciola hepatica* infection. Only adult animals (>2 years) were included. To ensure that all CE samples were from late stage of infection, only echinococcal cysts larger than 1.5 cm that were found in liver and/or lungs were sampled and transported in iced boxes within 2 hours to the laboratory for further examination. To determine cyst fertility, they were inspected through direct microscopy for the presence of protoscoleces. From each individual cyst, protoscoleces and/or germinal layers were subjected to genotypification through PCR-restriction fragment length polymorphism analysis using a 444-bp fragment of the cytochrome c oxidase subunit 1 (*cox1*) and the enzyme *AluI*, followed by PCR products sequencing to confirm *Echinococcus granulosus* infection [29]. Only samples from *Echinococcus granulosus sensu stricto* were included in the study. Fascioliasis diagnosis was made either by direct visualization of adult parasites in bile ducts or by signs of chronic infection, as previously described [36]. Cattle were considered co-infected when *Echinococcus granulosus* and *Fasciola hepatica* were found in the same individual, not necessarily the same organ.

During bleeding blood samples from infected and control animals were collected from the jugular vein in tubes added with a clot activator gel. Samples were kept refrigerated and transported in iced boxes within two hours to the laboratory. The samples were centrifuged at 1,000x*g* for 10 minutes, the serum was aspirated and stored at -20°C until analyzed.

### Cytokine quantification

Serum samples were prepared for analysis in a 96-well plate utilizing a Milliplex MAP Porcine Cytokine/Chemokine Magnetic Bead Panel (Millipore Corp., Billerica, MA) following the kit-specific protocols provided by Millipore to detect IFN-γ, IL-1, IL-2, IL-4, IL-6, IL-10, IL-12 and IL-18. We validated the use of this kit by comparing the amino acidic sequence between cattle and porcine cytokines; the similarity (%) was determined for IFN-γ (87.87%) (NM_174086; NM_213948), IL-1 (79.98%) (NM_174092; NM_214029), IL-2 (84.3%) (AF348423.1; JN851821.1), IL-4 (83.67%) (NM_173921.2; NM_214123.1), IL-6 (84.25%) (NM_173923.2; NM_214399.1), IL-10 (83.36%) (NM_174088.1; L20001.1), IL-12 (91.13%) (NM_174355.2; NM_213993.1) and IL-18 (90.67%) (NM_174091.2; AF191088.1). Analytes were quantified using a Magpix analytical test instrument, which utilizes xMAP technology (Luminex Corp., Austin, TX), and xPONENT 4.2 software (Luminex). The results were expressed as ng/mL.

### Study groups

In an initial analysis, cattle were classified as follows: Control "Ctrl" (n = 13), Cystic Echinococcosis "CE" (n = 47), Fascioliasis "FH" (n = 19) and, Cystic Echinococcosis with Fascioliasis "CE+FH" (n = 28). Control animals consisted in cattle without macroscopic parasite infections and both normal hemogram and biochemical profiles. The CE group was further divided according to their *Echinococcal* cysts fertility status either having fertile or infertile cysts then labeled as “CE fer” or “CE inf”.

### Statistical analysis

Data was entered into a Microsoft Excel 2010 database, followed by analysis carried out with SPSS v.19 for Windows (SPSS Italia SRL, Bologna, Italy) and Prism 6 software (Graphpad Software 6.0, San Diego, USA). Medians and interquartile ranges (IQR) were calculated for continuous measures. The Kruskal-Wallis test and Mann-Whitney U test were used for comparisons among several groups or pairwise comparisons, respectively. A statistically significant association between variables was considered to exist if the p value was below the 0.05 threshold.

## Results

Only cattle affected with *Echinococcus granulosus sensu stricto* were included in the present study because they represented 98% of all the analyzed cysts. Cytokine concentration was measured in a total of 107 animals, which met the inclusion criteria, and divided according to their infection status as follows: Ctrl (n = 13), CE (n = 47), FH (n = 19), CE+FH (n = 28). *Echinococcal* cyst fertility status resulted in CE Fer (n = 22) and CE Inf (n = 25). All cytokine concentration results are expressed as mean SD.

**Fig 1:**
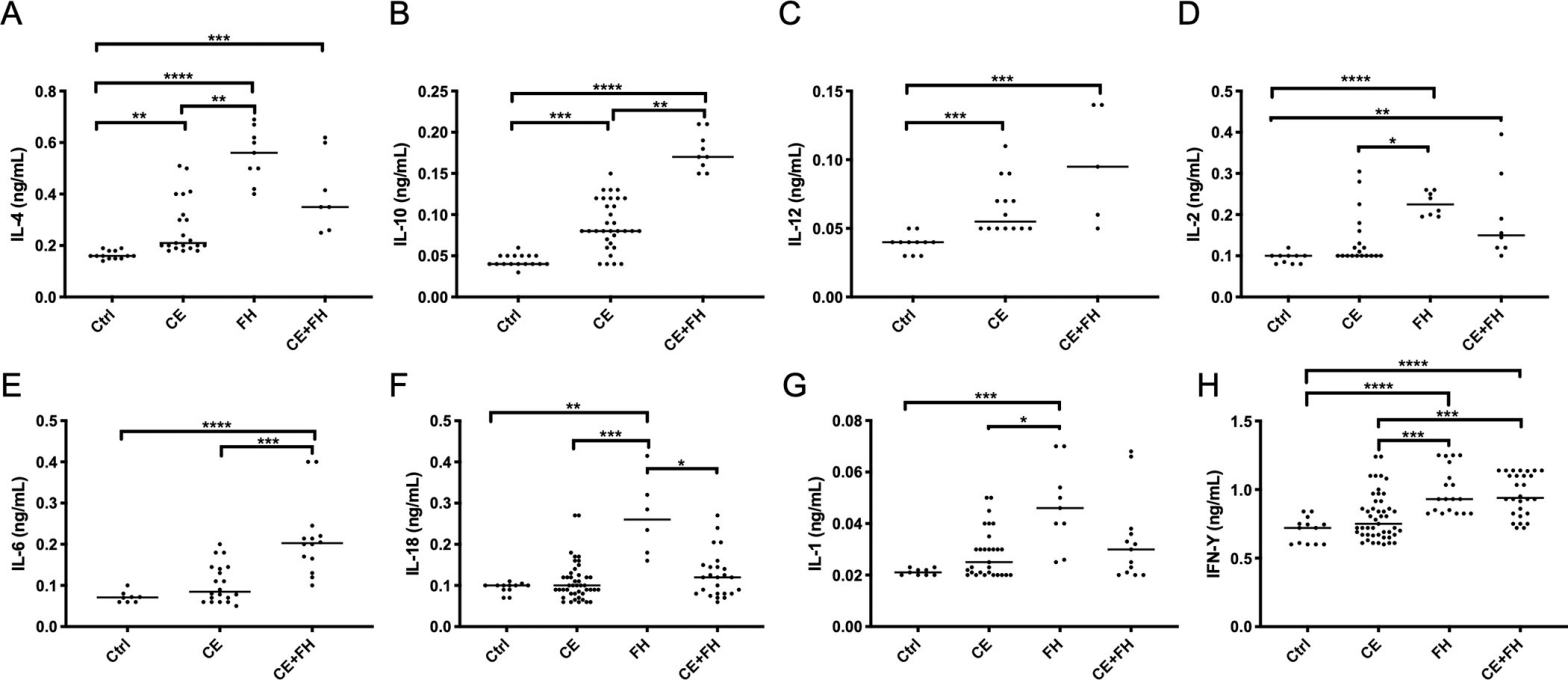
Serological cytokine concentration in cattle. Animals are divided in Control (Ctrl), Cystic Echinococcosis (CE), Fascioliasis (FH) and, co-infected animals (CE+FH). Cytokine concentrations for IL-4 (A), IL-10 (B), IL-12 (C), IL-2 (D), IL-6 (E), IL-18 (F), IL-1 (G) and IFN-γ (H) are shown. Dots represent individual cytokine values in cattle and the horizontal bar represents the mean for each group. Statistical significance was tested between groups using the Kruskal-Wallis test, with * = p < 0.05, ** = p< 0.01 and *** = p< 0.001.

The control group was used as baseline and fold changes of cytokine levels calculated with respect to this group. The highest concentration of serological IL-4 was observed in the FH group with a 3.43-fold increase, followed by CE+FH with a 2.5-fold increase and CE with a 1.68-fold increase. Significant differences were observed between all groups, with the higher significance found in FH group with respect to Ctrl group (Fig 1A). For IL-10, the highest concentration was observed in the CE+FH group with a 4.25-fold increase, followed by CE with a 2-fold increase. Significant differences were observed between infected groups, with CE+FH groups with higher significance with respect to Ctrl group (Fig 1B). For IL-12, the highest concentration was observed in the CE+FH group, which represented a 3-fold increase followed by CE with a 2-fold increase. Significant differences were observed between control and infected animals group (Fig 1C). For IL-2, the highest concentration was observed in the FH group with a 2.4-fold increase, followed by 2.1-fold increase in CE+FH and 1.4-fold increase in CE. High significance was observed between FH and Ctrl groups (Fig 1D). For IL-6, the highest concentration was observed in the CE+FH group with a 3-fold increase, followed by a 1.42-fold increase in CE group. Significant differences were observed between both infected groups, with CE+FH group with high significance (Fig 1E). For IL18, the highest concentration was observed in the FH group with a 2.8 fold increase, followed by CE+FH with a 1.33 fold increase and CE with a 1.22 fold increase. The higher significance was found between FH and CE infected animals (Fig 1F). For IL-1, the highest concentration was observed in the FH group with a 2-fold increase, followed by a 1.5-fold increase in both CE+FH and CE infected animals. High significance was observed between FH and Ctrl groups (Fig 1G). Finally, for IFN-γ, the highest concentration was observed in the FH group with 1.41-fold increase, followed by a 1.37-fold increase in CE+FH and 1.14-fold increase in CE infected animals. Significant differences were observed between all groups, with the higher significance in FH and CE+FH groups (Fig 1H).

**Fig 2:**
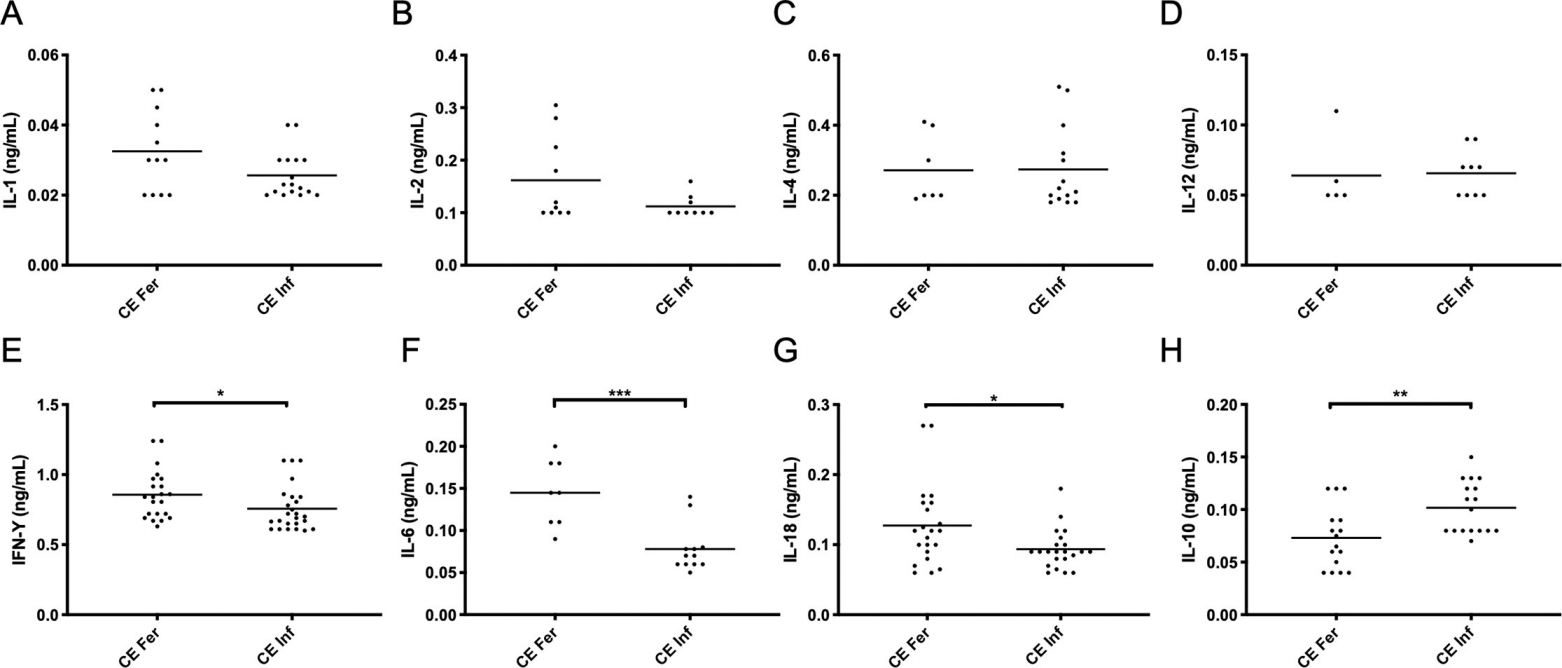
Comparison of serological concentration between fertile and infertile *Echinococcal* cysts. Animals are grouped in Cystic Echinococcosis with fertile cysts (CE Fer) and Cystic Echinococcosis with infertile cysts (CE Inf) for IFN-γ (a), IL-1 (b), IL-2 (c), IL-4 (d), IL-6 (e), IL-10 (f), IL-12 (g) and IL-18 (h). Dots represent individual cytokine values in cattle and the horizontal bar represents the mean for each group. Statistical significance was tested between groups using the Mann-Whitney U-test, with * = p < 0.05, ** = p< 0.01 and *** = p< 0.001.

When the fertility status of cysts of infected cattle was considered, analysis of serological IL-1 (Fig 2A), IL-2 (Fig 2B), IL-4 (Fig 2C) and IL-12 (Fig 2D) showed no statistical difference between cytokine levels of CE Fer and CE Inf groups (0.03±0.01ng/mL vs Inf 0.02±0.006ng/mL), (0.16±0.08ng/mL vs 0.11±0.02ng/mL), (0.27±0.09ng/mL vs 0.27±0.11ng/mL) and (0.06±0.02ng/mL vs 0.06±0.01ng/mL), respectively. A 1.13-fold for IFN-γ (Fig 2E), two-fold for IL-6 (Fig 2F) and 1.33-fold IL-18 (Fig 2G) increase were found in CE Fer when compared to CE Inf (0.85±0.17ng/mL vs 0.75±0.16ng/mL), (0.14±0.03ng/mL vs 0.07±0.02ng/mL) and (0.12±0.05ng/mL vs 0.09±0.02ng/mL), respectively, with higher significance in IL-6. Finally, there was a 1.42-fold increase of IL-10 (Fig 2H) in CE Inf when compared to CE Fer, with 0.10±0.02ng/mL vs 0.07±0.02ng/mL, respectively.

Overall, cattle in the FH group presented the highest cytokine concentrations, followed always by the CE+FH group. When considering fertility status, fertile *Echinococcal* cysts showed higher serological concentrations of IFN-γ, IL-6 and IL-18, while having lower IL-10 concentrations, as compared to serological concentrations of cattle harboring infertile *Echinococcal* cysts.

Due to samples being below the detection level of the kit, values for IL-6, IL-10 and IL-12 single infected with *Fasciola hepatica* are not available.

## Discussion

To the best of our knowledge, this is the first study dealing with serological cytokine production from late CE infection in cattle, and their relationship with cyst fertility or other parasitic co-infections.

Several studies show that both Th1 and Th2 cytokines are induced during CE. Cytokine production depends on the clinical stage of the disease, localization of the cysts, and pharmacological or surgical treatment [40–42]. Our study determines and compares the serological concentration of many circulating cytokines in control, single infected and co-infected cattle. We provide evidence that many circulating cytokine levels are higher in cattle infected with *Fasciola hepatica* compared to those co-infected with *E*. *granulosus sensu stricto*, with single CE infection or control animals.

Immunological studies in humans show elevated *in vitro* production of parasite antigen-driven Th1 (IFN-γ, IL-6), Th2 (IL-4, IL-5) and Treg (IL-10) cytokines by peripheral blood mononuclear cells isolated from patients with CE confirming that human immune responses to *E*. *granulosus* metacestode is regulated by a mixed Th1/Th2/Treg response [23]. Our results also demonstrate this occurrence in cattle. Data in humans indicate that in CE, mixed Th2/Treg responses, correlate with susceptibility to disease (active cysts) whereas a Th1 responses correlate with protective immunity (in-active cysts) [43].

IL-12 functions through the up-regulation of IFN-γ [44–46]. IFN-γ is shown to have *in vitro* anti-*Echinococcus* activity. IFN-γ also enhances the production of IL-12, creating a positive reinforcement loop which enhances Th1 type immunity [46]. IL-12 was originally termed ‘natural killer cell stimulatory factor’ or ‘cytotoxic lymphocyte maturation factor’. This is produced mainly by activated macrophages/monocytes. It has a very important role in the initiation and regulation of the innate cellular immune responses [44, 45, 47]. Interestingly, IL-12 levels were higher in CE only infected cattle compared to healthy animals, but not between the fertile and infertile CEs. We expected that infertile CEs would have higher levels of IL-12 cytokine as previous data on the role of cytokines in host anti-*Echinococcus* defense suggests that this cytokine underscores the ability of the metacestode to trigger cytokine production [41, 48–51].

IFN-γ is also a key cytokine that triggers the activation of macrophage function which, through nitric oxide (NO) production, inhibit the growth and function of helminths and other infectious agents, playing a relevant role in the establishment of protective Th1-mediated immunity during *E*. *granulosus* infection [50]. Elevated levels of both NO and IFN-γ are found *in vitro* and *in vivo* during human CE infections. Re-infected patients do not show detectable levels of either molecule [41, 48, 49]. Previous *in vitro* studies suggest that protoescoleces provide an activation signal, triggering NO induction in peripheral blood mononuclear cells from human patients and healthy donors. This reflects a complex host-parasite interaction. Our results were not in agreement with what was found for humans, as CE single infected animals did not show higher levels of the cytokine; this could be due to differences in the cellular immune response between cattle and humans. Significant differences were found between fertile cysts an infertile cysts where fertile cysts had slightly higher levels.

Reports show that CE patients with active cysts have a predominant Th2 profile, characterized by IL-4 production [10, 52, 53], and *in vitro* assays show that IL-4 reduces protoescolex killing, promoting parasite survival [54]. Previous work has shown that levels of IL-4 and IL-10 increased after *Echinococcus* infection in humans and experimental mouse models [11, 55]. These studies suggest that IL-4/IL-10 impairs the Th1 protective response and allows the parasite to survive in infected patients [40, 56]. Our study shows that IL-4 /IL-10 levels increase in CE infected cattle. However, IL-4 levels between cattle with fertile cysts and infertile cysts showed no significant differences.

The regulatory and anti-inflammatory cytokine IL-10 is abundantly expressed by leukocytes in CE infected hosts, especially in the immediate vicinity of the parasite [11, 57]. Systemic IL-10 levels increase in cattle with infertile cysts. This does not follow with the inflammatory response found locally. The adventitial layer of infertile cysts usually feature a strong innate inflammatory response consisting of a granulomatous reaction with palisading macrophages, including infiltration and disorganization of the laminated layer. Fertile cysts usually have a fibrotic local immune response [24]. Further studies on the expression of IL-10 in the adventitial layer of fertile and infertile ECs are needed to complete this data, as it is plausible that the granulomatous reaction of infertile ECs is concomitant with IL-10 expression.

In mouse experimental infections, the cytokine response during early stage of experimental infection by *E*. *granulosus* depends on the parasite dosage: a low dose induces a regulatory cytokine response while a high dose of parasites induces a type-2 cytokine response [58].

Cattle become infected with *F*. *hepatica* and *E*. *granulosus* while grazing. *Echinococcus granulosus* and *Fasciola hepatica* co-infection is present in almost 10% of cattle [36]. Since both parasites are able to survive for more than a decade in its mammalian host [6, 39], the chronic inflammatory response should also be simultaneous. Conversely, cattle that are slaughtered in abattoirs are usually treated for *Fasciola hepatica* infection during their productive life cycle, which eliminates the adult and migrating forms. In this scenario, it is plausible that adult co-infected cattle harbor both acute *F*. *hepatica* infections and chronic *E*. *granulosus* infections.

Cattle with Fascioliasis only, had significantly higher serological levels of IFN-γ, IL-1, IL-2, IL-4, and IL-18 than control and CE single infected cattle. IL-6, IL-10, and IL-12 were not obtained for this group however these cytokines were significantly higher in co-infected animals compared to healthy animals. IL-6, IL-10, and IFN-γ levels were higher in co-infected cattle than CE only. There were no differences found with respect to cytokines IL-12, IL-6 and IFN-γ between control and CE single infected animals. When FH co-infection was present, the cytokine levels were higher.

Since our results are obtained from naturally infected cattle, there are some limitations to our study, such as insecticide treatment during feeding, the temporality of *F*. *hepatica* and *E*. *granulosus* infection and use of antiparasitic drugs. However, since cattle did not show signs of other systemic diseases most of the confounding factors have been addressed.

To conclude, our results show that in naturally infected cattle, systemic cytokine profiles follow a distinct pattern between control, FH, CE and CE+FH: the highest concentrations are found when cattle are infected only with *F*. *hepatica*; these systemic concentrations decrease following the order CE+FH, CE and control animals.

## Supporting information

S1 Checklist (DOCX)

S1 File Serological concentrations (ng/mL) for IL-4, IL-10, IL-12, IL-2, IL-6, IL-18, IL-1 and IFN-γ from Control, Single infected and co-infected cattle. (XLSX)

S2 File Serological concentrations (ng/mL) for IL-1, IL-2, IL-4, IL-12, IFN- γ, IL-6, IL-18 and IL-10 from cattle with fertile and infertile echinococcal cysts. (XLSX)
