# Supplementary material for: Cattle co-infection of Echinococcus granulosus and Fasciola hepatica results in a different systemic cytokine profile than single parasite infection
Source: PLoS One. 2020 Sep 11;15(9):e0238909. doi: 10.1371/journal.pone.0238909 (PMC7485845; doi:10.1371/journal.pone.0238909)

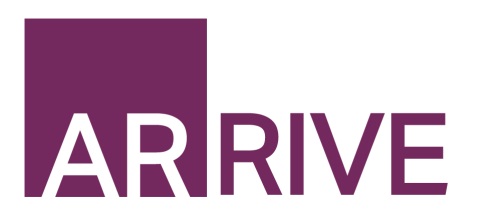


The ARRIVE Guidelines Checklist

Animal Research: Reporting In Vivo Experiments

Carol Kilkenny^1^, William J Browne^2^, Innes C Cuthill^3^, Michael Emerson^4^ and Douglas G Altman^5^

*^1^The National Centre for the Replacement, Refinement and Reduction of Animals in Research, London, UK, ^2^School of Veterinary Science, University of Bristol, Bristol, UK, ^3^School of Biological Sciences, University of Bristol, Bristol, UK, ^4^National Heart and Lung Institute, Imperial College London, UK, ^5^Centre for Statistics in Medicine, University of Oxford, Oxford, UK.*

|  | | ITEM | RECOMMENDATION | Section/ Paragraph |
| --- | --- | --- | --- | --- |
| 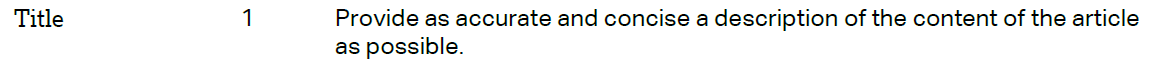 | | | Title |  |
| 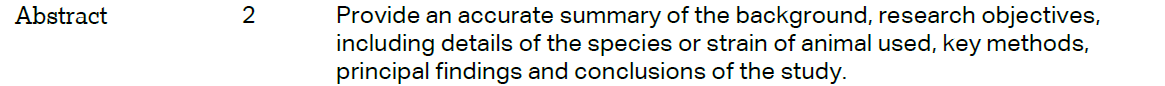 | | | Abstract |  |
| INTRODUCTION | | |  |  |
| 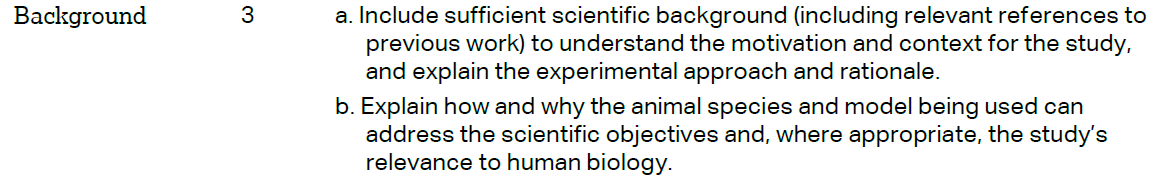 | | | Paragraphs 1-8 |  |
| 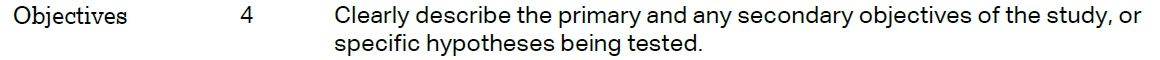 | | | Paragraph 9 |  |
| METHODS | | |  |  |
| 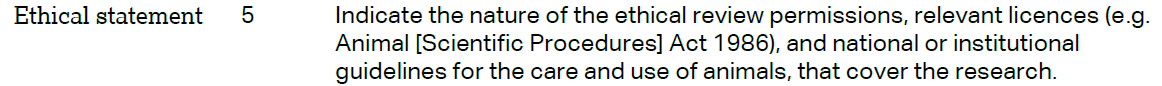 | | | Paragraph 1 |  |
| 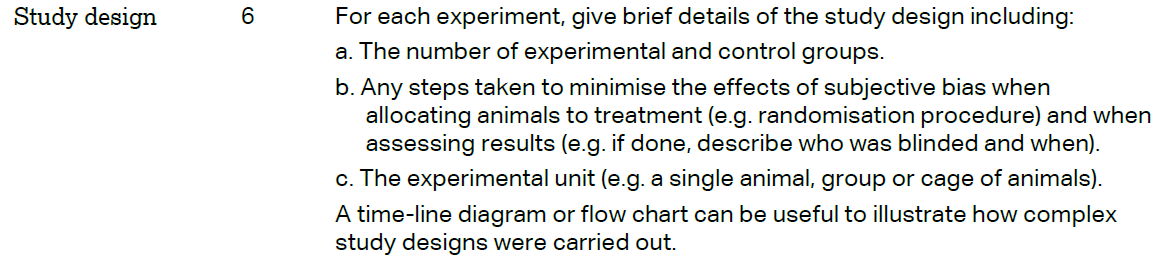 | | | Paragraph 4 |  |
| 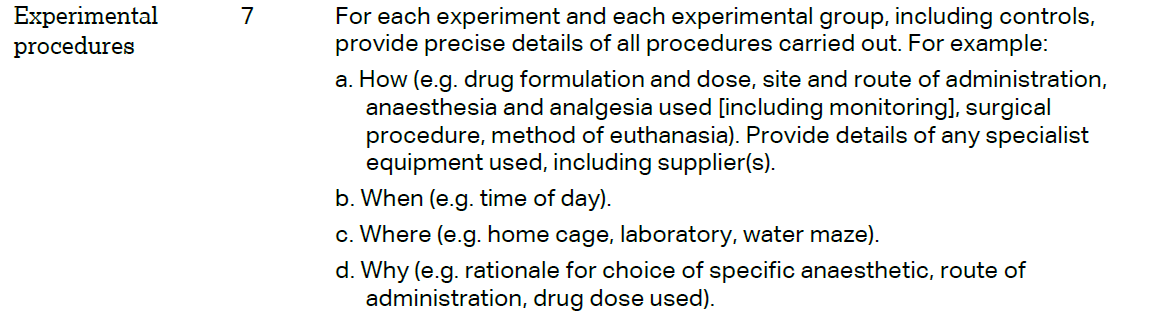 | | | Paragraphs 1-2 |  |
| 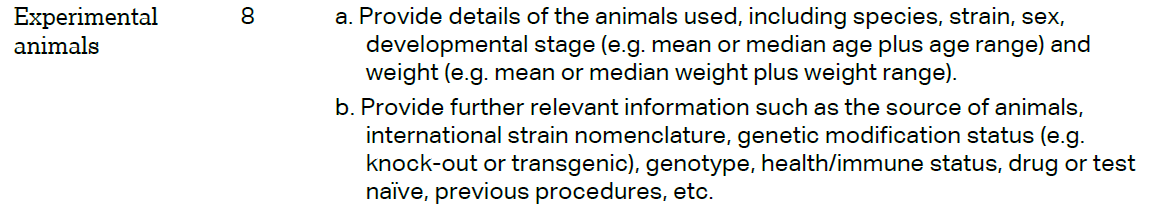 | | | Paragraph 1 |  |

The ARRIVE guidelines. Originally published in *PLoS Biology*, June 2010^1^

| 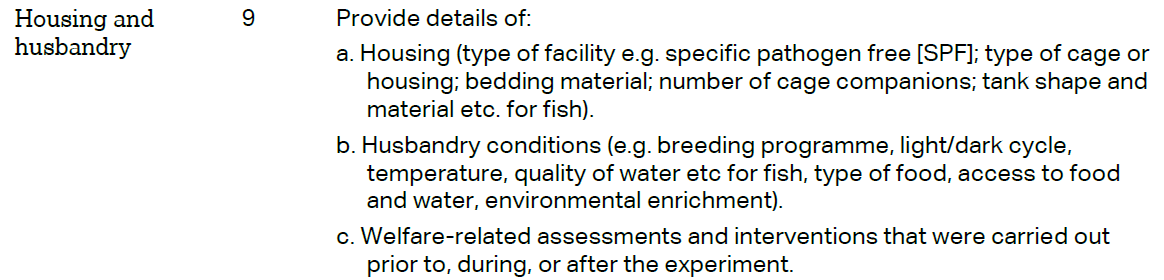 | Paragraph 1 | |
| --- | --- | --- |
| 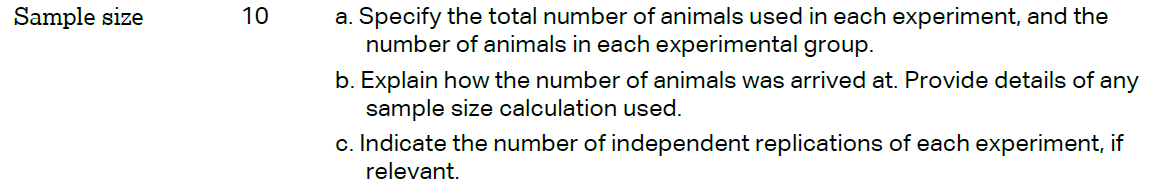 | Paragraph 4 | |
| 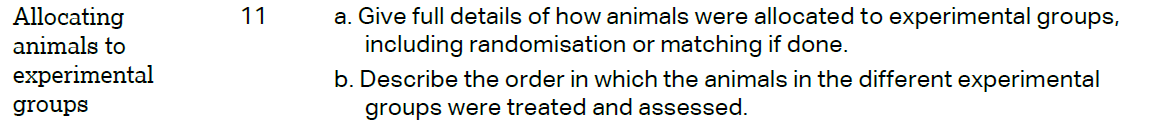 | Paragraph 4 | |
| 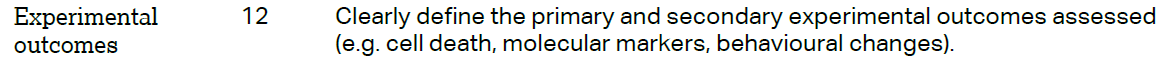 | Paragraph 3 | |
| 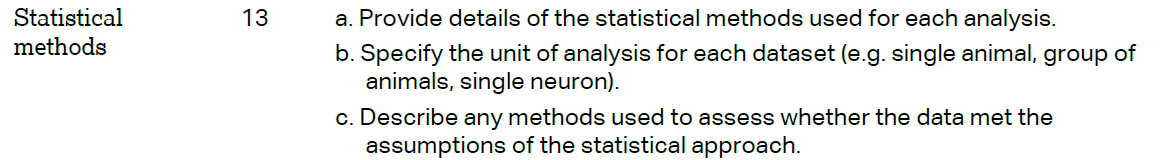 | Paragraph 5 | |
| RESULTS |  | |
| 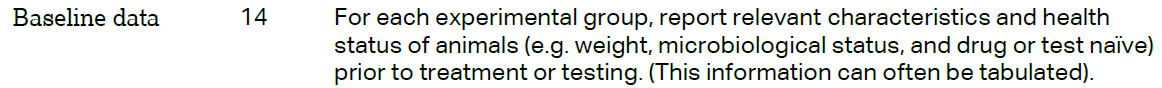 | Paragraph 1 | |
| 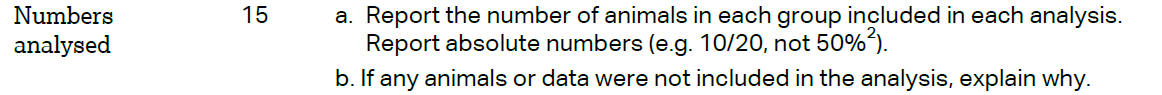 | Paragraph 1 | |
| 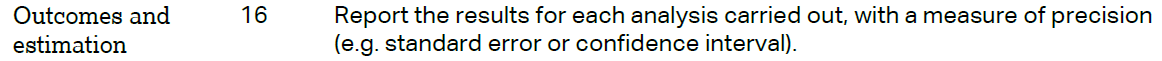 | Paragraph 2 | |
| 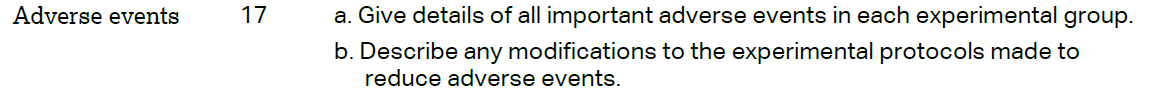 | Paragraph 5 | |
| DISCUSSION |  | |
| 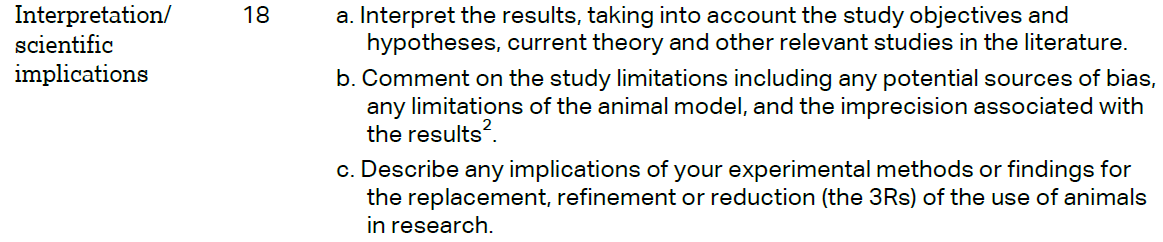 | Paragraphs 3-7, 9-10 | |
| 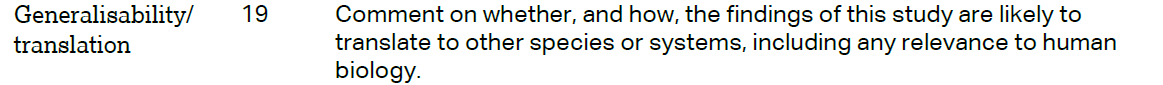 | Paragraphs 5-6 | |
| 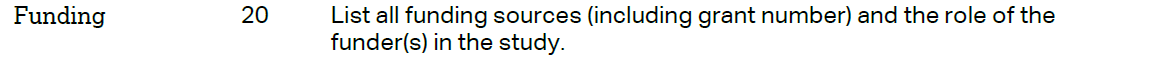 | | Paragraph 12 |


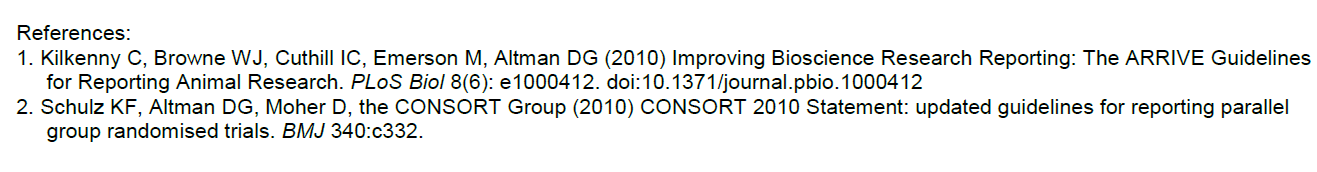

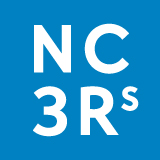

Supplement: S1 Checklist — (DOCX) [file pone.0238909.s001.docx]
